# Prediction of in-hospital mortality in patients on mechanical ventilation post traumatic brain injury: machine learning approach

**DOI:** 10.1186/s12911-020-01363-z

**Published:** 2020-12-14

**Authors:** Ahmad Abujaber, Adam Fadlalla, Diala Gammoh, Husham Abdelrahman, Monira Mollazehi, Ayman El-Menyar

**Affiliations:** 1 Assistant Executive Director of Nursing, Hamad Medical Corporation, Doha, Qatar; 2 Management Information Systems, Business, and Economics Faculty, Qatar University, Doha, Qatar; 3 Industrial Engineering, University of Central Florida, Orlando, USA; 4 Department of Surgery, Trauma Surgery, Hamad Medical Corporation, Doha, Qatar; 5 Department of Surgery, Trauma Surgery, Clinical Research, Hamad Medical Corporation, Doha, Qatar; 6 Department of Clinical Medicine, Weill Cornell Medical College, Doha, Qatar

**Keywords:** Traumatic brain injury, Machine learning predictive model, Mechanical ventilation, Mortality

## Abstract

**Background:**

The study aimed to introduce a machine learning model that predicts in-hospital mortality in patients on mechanical ventilation (MV) following moderate to severe traumatic brain injury (TBI).

**Methods:**

A retrospective analysis was conducted for all adult patients who sustained TBI and were hospitalized at the trauma center from January 2014 to February 2019 with an abbreviated injury severity score for head region (HAIS) ≥ 3. We used the demographic characteristics, injuries and CT findings as predictors. Logistic regression (LR) and Artificial neural networks (ANN) were used to predict the in-hospital mortality. Accuracy, area under the receiver operating characteristics curve (AUROC), precision, negative predictive value (NPV), sensitivity, specificity and F-score were used to compare the models` performance.

**Results:**

Across the study duration; 785 patients met the inclusion criteria (581 survived and 204 deceased). The two models (LR and ANN) achieved good performance with an accuracy over 80% and AUROC over 87%. However, when taking the other performance measures into account, LR achieved higher overall performance than the ANN with an accuracy and AUROC of 87% and 90.5%, respectively compared to 80.9% and 87.5%, respectively. Venous thromboembolism prophylaxis, severity of TBI as measured by abbreviated injury score, TBI diagnosis, the need for blood transfusion, heart rate upon admission to the emergency room and patient age were found to be the significant predictors of in-hospital mortality for TBI patients on MV.

**Conclusions:**

Machine learning based LR achieved good predictive performance for the prognosis in mechanically ventilated TBI patients. This study presents an opportunity to integrate machine learning methods in the trauma registry to provide instant clinical decision-making support.

## Background

More than 70 million persons are expected to suffer TBI per year globally [1]. Compared to other injuries, TBI leads to the highest mortality and permanent disability rates [2, 3]. Mortality in TBI is known to be highly associated with the severity of the TBI and the patient`s age [4]. Severe TBI is one of the common causes for the use of mechanical ventilation (MV) [5, 6]. Although MV is a common intervention in the intensive care units and has saved countless lives since it was first used in 1950s [7], patients receiving MV are prone to several complications and have higher mortality rate compared to other patients [8].

The prediction of the in-hospital mortality in early stage following the TBI is crucial. The powerful and early prediction may help guide the clinicians to initiate the appropriate diagnostics and interventions in a timely fashion and provide better guidance to the patients` families. It also helps the healthcare managers to devote the optimal resources that are required to achieve the goals of the treatment plans [2, 9]. Nevertheless, predicting the prognosis of a disease requires developing sound prognostic models which utilize adequately large sample and attain a high degree of internal and external validity to be generalizable beyond the specific research contexts [10].

Glasgow-Coma Scale (GCS) is widely used to guide the clinicians` treatment decisions and to predict disease outcomes [2, 11]. Nonetheless, it is undeniable that GCS can be impacted by various factors e.g. alcohol intoxication that negatively impacts the prediction model`s accuracy and discrimination power. Therefore, it is crucial to consider several risk factors jointly such as age, injury characteristics, GCS and others when designing prognosis prediction model in order to enhance the model`s performance [11–13]. Over the past few decades, there were several published prognostic models. However, only few satisfied the sample size and validity requirements [11]. None of these models is designed exclusively to predict the mortality in TBI patients who receive MV. For example, Trauma Injury Severity Score (TRISS) aims to calculate the probability of survival and the outcomes in admitted trauma patients with or without TBI or MV [14]. Similarly, The International Mission for Prognosis and Analysis of Clinical Trials in TBI (IMPACT), Marshal Scale, Helsinki CT score, Corticosteroid Randomization After Significant Head injury (CRASH) and Rotterdam CT score are all prognostic models that aim to predict mortality in adult patients with TBI but are not exclusive for patients with moderate to severe TBI who received MV.

Researchers have published various prognostic models which aimed to help clinicians to predict the outcomes and prognosis following TBI. In 2013, Jacobs and colleagues [15] presented a model that predicted moderate to severe TBI outcomes. They used patients` demographic characteristics; brain CT scan findings and other clinical data such as vital signs, pupil’s reaction and GCS score as independent variables. Age, pupil responses, GCS score and hypotension following the injury in addition to the CT scan`s findings were found to be significant predictors for the TBI outcomes. On the other hand, there is a significant growing interest in the machine learning techniques during the last decade. Various recent studies provide evidence that the prognostic tools that employ machine learning approaches are more powerful than the prognostic tools that utilize the classical multivariate techniques [7, 16].

Senders et al., [17] systematically reviewed 30 publications that utilized machine learning approaches to predict mortality and other neurological outcomes following TBI. They concluded that machine learning based prognostic models outperformed many of the well-known predictive tools that use the conventional analytical techniques. Further, they found that machine learning models perform better than or at least similar to the field experts in some scenarios. Rau and colleagues [9] used age, gender, GCS, vital signs, co-morbidities and the use of helmet to design a machine learning model that predicts post moderate to severe TBI mortality. The authors compared the performance of several machine learning models (logistic regression (LR) and Artificial Neural Network (ANN)) using several performance metrics (i.e. accuracy, sensitivity, specificity and Area Under the Curve (AUROC)). ANN outperformed the other models with AUROC of 96.8%, accuracy of 92%, sensitivity of 84.4% and specificity of 92.8%.

In a similar vein, another study conducted on 565 pediatric patients that suffered TBI, Hale et al. [18] designed ANN based model to predict several outcomes including mortality. GCS score, pupillary reaction, brain CT scan findings, in addition to blood glucose level and hemoglobin concentration were used as predictors. Interestingly, they compared the performance of the machine learning model with Helsinki, Rotterdam, and Marshall prognostic models and found that the machine learning model not only achieved a high discrimination power (AUROC > 94%), but also it outperformed the three models. This was also supported by Eftekhar et al. [16] who reported that ANN significantly outperformed the models that use logistic regression in disease outcomes prediction with AUROC of 96.5% vs. 95.4%.

Through utilizing the trauma registry data, this study aims to introduce machine learning based prognostic model that helps clinicians predict the in-hospital mortality in adult patients who received mechanical ventilation after sustaining moderate to severe TBI.

## Methods

A retrospective analysis was conducted for all adult patients who sustained TBI and were hospitalized in the trauma center from January 2014 to February 2019 with an abbreviated injury severity score for head region (HAIS) ≥ 3. The Trauma center is a part of Hamad Medical Corporation (HMC) network which is a governmental non-for-profit healthcare organization.

**Fig. 1:**
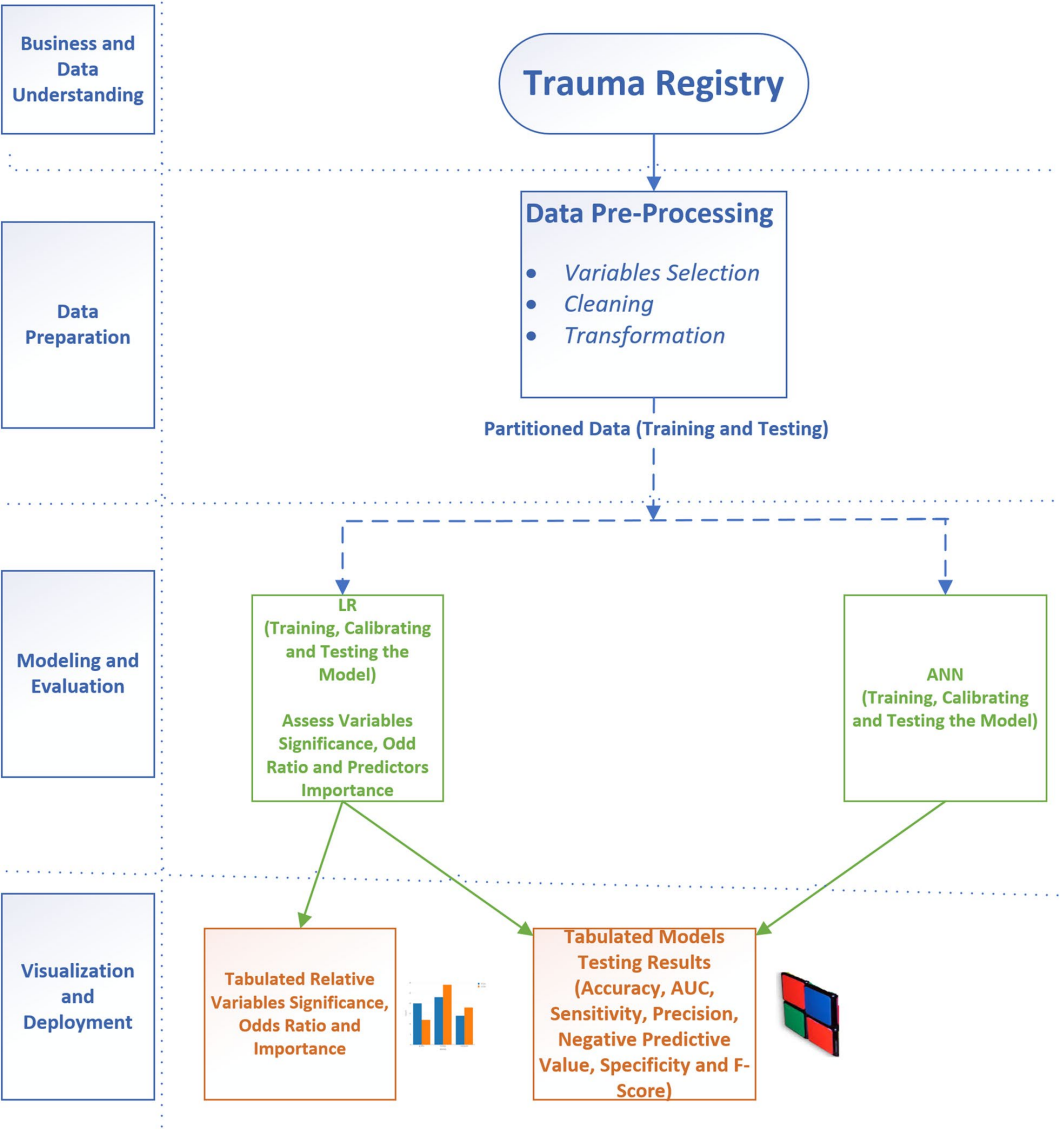
Summarizes the research methodology

The study is compliant with the Cross-Industry Standard Process for Data Mining (CRISP-DM). The CRISP-DM provides a framework that identifies six phases to the data mining projects; business and data understanding, data preparation, modeling, evaluation and deployment [19] (Fig. 1).

### Business and data understanding

The trauma registry involves enormous number of variables where some are not usable in this research. Accordingly, to understand the trauma registry data, we referred to the trauma registry data dictionary to explore the definition of every variable. We also consulted the past literature to determine which variables to be considered predictors and which among them to impute if they have missing values [20].

We excluded pediatric patients (< 14 years old) to facilitate the understanding and the interpretability of the findings especially that some key variables are interpreted differently between the adult and pediatric age groups (e.g. vital signs). Abbreviated Injury Score for head region (HAIS) was used to determine the severity of TBI. HAIS 3 and 4 were considered moderate severity while HAIS 5 was considered severe TBI [21, 22]. Further, many patients in the sample sustained multiple system injuries. Therefore, patients who sustained trauma in other body regions that score AIS greater than the HAIS (12 patients) were excluded to ensure that the main injury of concern is the TBI.

These 12 patients were later included in a secondary analysis to test the rigor of the predictive model and whether adding them will significantly affect the model`s performance. Out the 12 patients, 5 had severe abdominal injury with one death, 3 had severe chest injury, 1 had severe spinal injury, and 3 had extremities injury.

### Data preparation

This research was approved by the Institutional Review Board (IRB# MRC-01–19-106) of the medical research center at HMC. The study targeted adults hospitalized in the trauma center at Hamad General Hospital following moderate to severe TBI and received mechanical ventilation and were captured in the trauma registry (N = 823).

Patients who sustained other systematic injuries with AIS > HAIS (12 recoreds) were excluded to ensure that the primary injury is TBI. Variables such as health record number, admission or disposition dates were not considered as they don’t have predictive power. Due to the serious impact of the missing data on the models` performance [23], literature suggests various methods to handle the missing data such as the elimination of incomplete records [9] or to carry out certain imputation methods [23]. Due to the critical nature of this study, we opted to eliminate the incomplete records (N = 26). This resulted in 785 eligible patient records for the final analysis.

### Outcome variable

The in-hospital mortality variable is binary (0 = survival and 1 = mortality). Patients who were discharged or transferred to other healthcare facilities were considered alive.

### Prediction models

The objective of this study is to develop a machine learning model that helps clinicians predict the in-hospital mortality in patients who received MV post moderate to severe TBI. Two supervised machine learning techniques (LR and ANN) were used to help provide performance comparative perspectives that enable the authors recommend the model which accomplishes better predictive performance and has higher chances to support the clinical decision making. We used SPSS modeler 18.1 to build the models and run the analysis.

Data were partitioned into three sets; training set 60%, validation set 20% and testing set 20%. Further, the overfit prevention was set at 30%. Table 1 shows the data partitions.

**Table 1:** Data partitions

Set	Proportion	Number of cases	Number of alive patients	Number of dead patients
Training set	70%	550	408 (74.2%)	142 (25.8%)
Testing set	30%	285	173 (60.7%)	62 (39.3%)
Total	100%	785	581 (74%)	204 (26%)

### Logistic regression (LR)

LR is a typical technique for predicting binary, binomial or multinomial outcomes [20]. It usually describes the relationship between a dichotomous dependent variable and a set of predictor variables that can be either numerical or categorical/dummy variables. Typically, LR is used for the prediction of the probability of the occurrence of an event by fitting data to a sigmoidal (S-shape) logistic curve. Usually, LR uses a numerical cutoff value (0.5). So, cases > 0.5 are classified (1 = success) and the rest are categorized (0 = failure) [24]. Thus, logistic regression is an appropriate procedure for predicting mortality in TBI patients who received MV. We utilized bi-directional step-wise LR to control the confounding variables effect [9].

### Artificial neural networks (ANN)

Although scholars considered ANN as a black-box machine learning tool, it is a widely used approach that is superior in solving classification and pattern identification problems [25]. It is also undeniable that ANN has a great capacity to support the clinical decision through engaging with the evidence-based medicine [18].

In this study, the ANN architecture was a standard feed-forward, back-propagation multi-layer perception (MLP) ANN. This MLP ANN consists of three layers; one input layer that had the study predictors, one hidden layer that consisted of 6 inaccessible neurons and one output layer. We opted to design ANN using the MLP as it performed better than the Radial Basis Function (RBF) during the initial assessment with accuracy and AUROC of 80.9% and 87.5%, respectively vs. 77.9% and 79.5%, respectively.

In the ANN and other machine learning methods, data are usually partitioned into training and test sets in order to optimize the model`s performance. Basically, the training lasts until the error cannot be further reduced [26]. Whenever training is completed, we can use the ANN to predict future instances with unknown outcomes [24]. One of the most important caveats in ANN is that it is prone to overfitting compared to LR. The reason is that the training makes the model perfectly fits the data set. Thus with new data sets, the prediction might be poor [24].

Ayer et al. [27] compared the two methods in several aspects: LR requires more statistical knowledge than ANN. However, ANN is more powerful in capturing complex relationships and determining interesting patterns in data. LR is easier to interpret and to identify the important predictors compared to the ANNs. The discrimination power and the prediction performance for both methods are good in general which makes it difficult to determine the superiority of one method over the other. Although the majority of studies compared the performance of the two methods reported that one of them outperformed the other, the performance in general was similar [16, 24, 27, 28].

## Results

In this study, 785 patients were included, of them 204 (26%) were deceased during their hospital course. The average age of the cohort was 33 years while it was 36.9 years for the deceased group. Motor vehicle crash (37.5%) was the most common mechanism of injury followed by fall from height (25.4%). Subdural hemorrhage (29%) followed by extradural hemorrhage (21%) were the most common CT findings. Almost one third of cases had midline shift (33.6%).

Refer to Tables 2 and 3 for the sample descriptive statistics.

**Table 2:** Sample characteristics- continuous variables

Variable	N	Mean	SD	Mean at death
Age	785	33	13.4	36.9
Injury severity score (ISS)	785	28.2	10.4	33.8
ED systolic blood pressure (SBP)	785	126.34	27.7	119
ED heart rate (HR)	785	102.8	25	107.7

**Table 3:** Sample characteristics—nominal and ordinal variables

Variable	Category	Count/%	With outcome 0 (alive)/%	With outcome 1 (dead)/%
Race	Asian	456/58.1	337/73.9	119/26.1
	Other	329/41.9	244/74.2	85/25.8
	Total/%	785/100	581/74.1	204/25.9
Mechanism of injury (MOI)	MVC	294/37.5	222/75.5	72/24.5
	Fall	199/25.4	142/71.4	57/28.6
	Pedestrian	162/20.5	110/67.9	52/32.1
	Other	130/16.6	107/82.3	23/17.7
	Total/%	785/100	581/74.1	204/25.9
Mode of arrival	Ambulance	639/81.4	455/71.2	184/28.8
	Other	146/18.6	126/86.3	20/13.7
	Total/%	785/100%	581/74.1%	204/25.9%
Multiple rib fractures	No	600/76.4%	454/75.7%	146/24.3%
	Yes	185/23.6%	127/68.6%	58/31.4%
	Total	785/100%	581/74.1%	204/25.9%
Lung contusion	No	509/64.8%	387/76%	122/24%
	Yes	276/35.2%	194/70.3%	82/29.7%
	Total	785/100%	581/74.1%	204/25.9%
Hemothorax	No	678/86.4%	514/75.8%	164/24.2%
	Yes	107/13.6%	67/62.6%	40/37.4%
	Total	785/100%	581/74.1%	204/25.9%
Pneumothorax	No	594/75.7%	456/76.8%	138/23.2%
	Yes	191/24.3%	125/65.4%	66/34.6%
	Total	785/100%	581/74.1%	204/25.9%
Midline shift	No	521/66.4%	416/79.8%	105/20.2%
	Yes	264/33.6%	165/62.5%	99/37.5%
	Total/%	785/100	581/74.1	204/25.9
TBI diagnosis/ CT findings	SDH	226/28.8	151/66.8	75/33.2
	EDH	161/20.5	140/87	21/13
	SAH	86/11	48/55.8	38/44.2
	CONT	119/15.2	101/84.9	18/15.1
	DAI	106/13.5	85/80.2	21/19.8
	Other	87/11.1	56/64.4	31/35.6
	Total/%	785/100	581/74.1	204/25.9
Cerebral edema	No	701/89.3	552/78.7	149/21.3
	Yes	84/10.7	29/34.5	55/65.5
	Total/%	785/100	581/74.1	204/25.9
Head AIS (HAIS)	3	241/30.7	218/90.5	23/9.5
	4	187/23.8	140/74.9	47/25.1
	5	357/45.5	223/62.5	134/37.5
	Total/%	785/100	581/74.1	204/25.9
Face AIS (FAIS)	0	399/50.8	276/69.2	123/30.8
	1	85/10.8	70/82.4	15/17.6
	2 (2–5)^a^	301/38.3	235/78.1	66/21.9
	Total/%	785/100	581/74.1	204/25.9
Chest AIS (CAIS)	0	353/45	282/79.9	71/20.1
	1 (1–2)^a^	120/15.3	82/68.3	38/31.7
	2 (3–5)^a^	312/39.7	217/69.6	95/30.4
	Total/%	785/100	581/74.1	204/25.9
Abdomen AIS (AAIS)	0	610/77.7	473/77.5	137/22.5
	1 (1–2)^a^	104/13.2	67/64.4	37/35.6
	2 (3–5)^a^	71/9	41/57.7	30/42.3
	Total/%	785/100	581/74.1	204/25.9
Spine AIS (SAIS)	0	538/68.5	402/74.7	136/25.3
	1 (1–5)^a^	274/31.5	179/72.5	68/27.5
	Total/%	785/100	581/74.1	204/25.9
Extremities AIS (EAIS)	0	416/53	316/76	100/24
	1 (1–2)^a^	262/33.4	194/74	68/26
	2 (3–5)^a^	107/13.6	71/66.4	36/33.6
	Total/%	785/100	581/74.1	204/25.9
Known comorbidities	No	659/83.9	496/75.3	163/24.7
	Yes	126/16.1	85/67.5	41/32.5
	Total/%	785/100	581/74.1	204/25.9
Intubation location	In-hospital	267/34	210/78.7	57/21.3
	Pre-hospital	518/66	371/71.6	147/28.4
	Total/%	785/100	581/74.1	204/25.9
VTE prophylaxis	No	180/22.9	60/33.3	120/66.7
	Yes	605/77.1	521/86.1	84/13.9
	Total/%	785/100	581/74.1	204/25.9
Blood transfusion	No	252/32.1	228/90.5	24/9.5
	Yes	533/67.9	353/66.2	180/33.8
	Total/%	785/100	581/74.1	204/25.9

MVC: Motor vehicle crash, SDH: subdural hemorrhage, EDH: epidural hemorrhage, SAH: subarachnoid hemorrhage, CONT: hemorrhagic contusion, DAI: diffuse axonal injury, VTE: venous thromboembolism

^a^Median and range

### Performance of the machine learning models

Table 4 demonstrates the models` performance in the test data partition. To obtain a comprehensive overview of the models’ performance, accuracy, AUROC, precision, negative predictive value, sensitivity, specificity and F-score measures were taken into consideration. LR achieved better performance than ANN with AUROC of 90.5% and accuracy of 87%.

**Table 4:** Performance of the classification models

Model	Number of predictors	Accuracy (%)	AUROC	Precision	Sensitivity	Specificity	F-Score
LR	8	86.8	92.00	0.82	0.65	0.95	0.72
ANN	24	85.5	91.40	0.76	0.66	0.92	0.71

Secondary analysis: The rigor of the model has been further tested by adding the 12 previously excluded records of patients who sustained injuries in areas that have AIS greater than the HAIS. The performance of the secondary model after adding the 12 records hasn’t changed significantly and resulted in the same set of predictors. The AUROC slightly reduced to 90.4%, accuracy of 86.4%, precision of 77%, negative predictive value of 88%, sensitivity of 62%, specificity of 94% and F-score of 69%. This presents a good indicator about the stability of the model. Nevertheless, to enhance the interpretability of the outcomes, we maintained the main model that excluded the 12 patients.

### In-hospital mortality risk factors

**Fig. 2:**
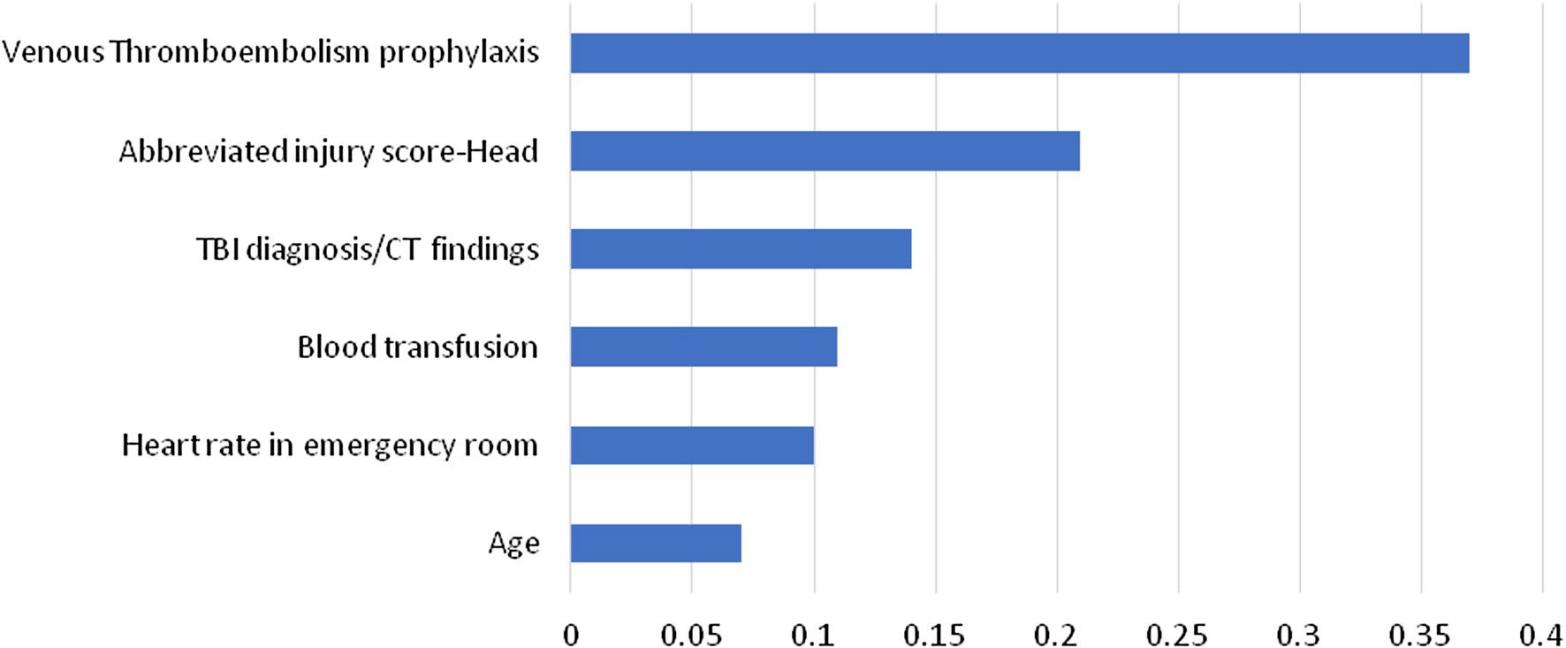
Predictors importance in logistic regression

LR identified 6 predictors (administration of VTE prophylaxis, HAIS, TBI diagnosis/CT finding, the need for blood transfusion during resuscitation, ED heart rate (HR) and age) as independent risk factors for the in-hospital mortality of the intubated patients with moderate to severe TBI (Table 5). The administration of VTE prophylaxis was ranked first in the predictor importance (0.37) followed by severity of head injury as measured by AIS (HAIS) (0.21). Figure 2 shows ranking of the important predictors.

**Table 5:** significant predictors estimate and likelihood ratio assessment

Predictor	B coefficient	*p* Value	EXP(B)
VTE (No)	3.5	< 0.05	33.12
VTE (Yes): reference			
TBI diagnosis/CT finding (EDH)	-1.501	< 0.05	0.223
TBI diagnosis/CT finding (Other): reference			
Cerebral edema (No)	-1.847	< 0.05	0.158
Cerebral edema (Yes): reference			
Blood transfusion (No)	-1.824	< 0.05	0.161
Blood transfusion (Yes): reference			
HAIS = 3	-2.033	< 0.05	0.131
HAIS = 5: reference			
Age	0.033	< 0.05	1.034
ED HR	0.025	< 0.05	1.026
Arrival mode (1 = Ambulance)	0.877	< 0.05	2.404
Arrival mode (2 = other): reference

**Fig. 3:**
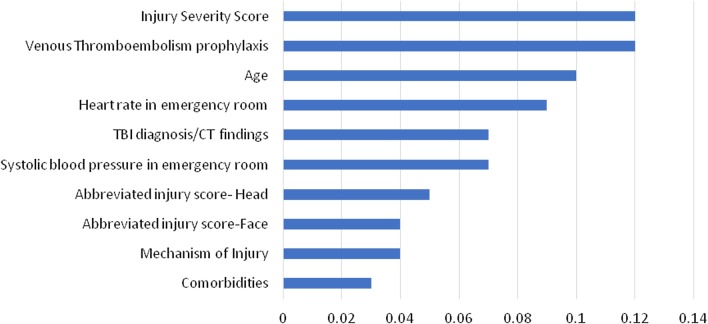
Predictors importance in artificial neural networks

On the other hand, ANN used all the 24 predictors to predict the in-hospital mortality. ANN achieved 80.9%accuracy and 87.5% AUROC with Injury Severity Score (ISS) ranked first in the predictor importance (0.12). Figure 3 ranks the top ten predictors based on their importance.

Predictor`s importance refers to the particular predictor`s contribution to the model`s performance. Usually, the predictors are ranked based on their contribution to the model capacity where the first predictor is the most important, then the remaining predictors are ranked relative to the first one [29] (Figs. 2 and 3).

## Discussion

In this analysis, we identified that the likelihood of mortality in TBI patients increased amongst patients who did not receive venous thromboembolism (VTE) prophylaxis relative to those who received VTE prophylaxis which is consistent with prior literature [30]. Around 67% of patients who did not receive VTE prophylactic agents have died compared to 13.9% of those who received the VTE prophylaxis and died. The odds of mortality given that a patient doesn’t receive VTE prophylaxis post-moderate to severe TBI increases by 31 folds compared to one who receives it. The previous literature reported a significant association between TBI and VTE [31, 32]. Although there is debate that administering VTE prophylaxis may increase the hemorrhagic risks in patients with TBI, data suggest that administering VTE prophylaxis in 24 to 48 h post TBI is safe following a proper risk assessment [30, 33]. Thus, we could argue that the non or delayed administration of VTE prophylaxis may lead to VTE events that may subsequently contribute to a higher risk of mortality. Therefore, early administration of VTE prophylaxis could improve TBI patients` prognosis and reduces the risk of coagulation-related mortality. Additionally, we observed that patients who sustained severer TBI (as measured by AIS) were more likely not to receive VTE prophylaxis (17.4% of those with HAIS 3 vs 29.4% with HAIS 5). This is consistent with Nathens et al. [34] who concluded that physicians are conservative in administering VTE prophylaxis for patients with severe TBI. We further evaluated if there is any association between a TBI diagnosis and administration of VTE prophylaxis and we found a significant association (X^2^ = 13.49; P < 0.05). Patients with cerebral edema were more likely not to receive VTE prophylaxis (34.5%) compared to 24.6%, 22.6%, 20.9%, 21.4% and 20% in patients with subdural hemorrhage, extradural hemorrhage, subarachnoid hemorrhage, brain contusions and diffuse axonal injury, respectively. It is important to note that AIS is not necessarily available for the treating physicians at the time of making the decision to administer VTE prophylaxis. Therefore, we argue that the perceived severity of TBI as per patient`s presentation and clinical examination could influence the physicians` decision. Importantly, this finding may support the argument that the relation between the VTE prophylaxis and mortality doesn’t reflect causality, but it is rather associated with the severity of injury.

It is widely accepted that the severer the head injury, the higher the probability of mortality and unfavorable outcomes [14, 15, 35]. This study proved that patients with higher HAIS (AIS 5) have higher likelihood of mortality compared to those with lower HAIS (AIS 3). Only 9.5% of the patients who had HAIS 3 have died compared to 25.2% and 37.5% of patients who had HAIS 4 and 5 respectively have died. The odds of mortality given that a patient has severe TBI (HAIS = 5) increase significantly by 88.4% compared to those with HAIS 3. We found no significant difference in the mortality between patients who had HAIS 4 and HAIS 5.

The TBI diagnosis as identified by the initial brain CT scan plays a role in determining patients who are at risk for in-hospital mortality. Cerebral edema is a secondary complication to TBI which is considered a key risk factor for the in-hospital mortality. More than 65% of patients who had cerebral edema have died compared to 31.4%, 31.3%, 18.3%, 11% and 9.2% of those who had subdural hemorrhage, subarachnoid hemorrhage, diffuse axonal injury, extradural hemorrhage and brain contusions; respectively. The odds of mortality given that a patient sustained cerebral edema are 7 times higher than patients with other TBI findings.

According to Jha et al. [36], the majority of patients who have post-TBI mass lesion suffer from cerebral edema as a secondary complication. This explains why the TBI management guidelines put great emphasis on the prevention of the secondary brain injury and on the maintenance of the adequate cerebral perfusion pressure (CPP) [37, 38].

Interestingly, it is found that patients who underwent blood transfusion have higher odds of in-hospital mortality compared to those who haven’t received blood during resuscitation. 33.8% of the patients that received resuscitative blood transfusion died compared to 9.5% of those who didn’t need blood transfusion during resuscitation. The odds of mortality given that a patient receives blood during resuscitation increase significantly by 80.9% compared to those who don’t require blood transfusion during resuscitation. The indications and the complications of the resuscitative blood transfusion in TBI are controversial. Many scholars found a significant association between the blood transfusion and various unfavorable outcomes in patients with TBI [39, 40]. One possible explanation could be that those who required resuscitative blood transfusion are the patients who sustained severe injuries that caused significant blood loss which is a reason for poor TBI outcomes. We argue that blood transfusion per say doesn’t have a direct causal relationship with the mortality. Nevertheless, the reasons that indicate the need for blood transfusion during resuscitation i.e. bleeding and hypovolemia could be considered predictors for mortality.

The patient HR upon arrival to the ED is an indicator of organ perfusion adequacy. Tachycardia (HR > 100 bpm), in patient with trauma, could be an indicator of hypovolemic shock that may negatively impact the CPP. This negative effect worsens when tachycardia is associated with low SBP (< 90 mmHg) which leads to poor TBI outcomes [38, 41]. The mean HR upon admission to the emergency room following TBI was 102.8 beats per min while the mean HR upon arrival for those who died was 107.7 beats per minute. An increase in HR by one unit may change the odds of mortality by approximately 3% (odds ratio = 1.028, P < 0.05).

Age was also found to play a significant role in predicting in-hospital mortality in patient with TBI who received MV [9]. An increase by one year of age increases the likelihood of mortality by more than 3.6%. (Odds ratio = 1.036, P < 0.05). The patients` mean age in this study was 33 years. However, the mean age of those who died during their hospitalization was 36.9 years.

### Limitations

The sample of 785 patients in five years is considered a small sample in the field of machine learning. The sample size of this study was challenging in several respects such as but not limited to the class imbalance, management of missing data and cross validation. However, we have to consider the relatively small population (2.8 million) of the country. Regional or international multicenter studies could help overcome this study limitation. Qatar national trauma registry has regular internal and external validation, moreover, the registry is abstracting data from the only tertiary level 1 trauma center in the country. Of note, some of the potentially important predictors such as time to surgical procedures and other unfavorable outcomes were not captured in the study data set. The availability of such variables may enhance the predictive performance and improve the clinical insight that can be obtained by this study.

## Conclusions

Although plenty of literature focuses on predicting mortality in TBI patients, there is a dearth of literature that aims to deploy machine learning techniques to predict in-hospital mortality in intubated patients post-TBI. Accordingly, this study is thought to provide a valuable contribution to this field of research. This study demonstrates that LR provides better performance than the ANNs in predicting in-hospital mortality for patients who received mechanical ventilation post moderate to severe TBI.

The study results are encouraging and provide an opportunity for integrating the machine learning methods with trauma registry and electronic health records. This would attain an instant clinical decision support to healthcare providers. In addition, with limited data size, machine learning algorithms demonstrate powerful predictive power which opens the door for integrating the artificial intelligence modalities with medical practice to enhance patient’s treatment outcomes.

## Data Availability

All data were shown in the results, tables and figures.

## Abbreviations

LR: Logistic regression
ANN: Artificial neural networks
TBI: Traumatic brain injury
AUROC: Area under the receiver operating characteristics curve
SVM: Support vector machine
